# Q Fever in Southern California: a Case Series of 20 Patients from a VA Medical Center

**DOI:** 10.4269/ajtmh.18-0283

**Published:** 2019-05-20

**Authors:** Christine M. Akamine, Mario L. Perez, Jea Hyun Lee, Michael B. Ing

**Affiliations:** 1 Department of Internal Medicine, Loma Linda University Health, Loma Linda, California;; 2 Division of Infectious Diseases, Kaiser Permanente Fontana Medical Center, Fontana, California;; 3 Infectious Diseases Section, Veterans Affairs Loma Linda Healthcare System, Loma Linda, California

## Abstract

Query fever (Q fever), caused by *Coxiella burnetii*, was first described in southern California in 1947. It was found to be endemic and enzoonotic to the region and associated with exposure to livestock. We describe a series of 20 patients diagnosed with Q fever at a Veterans Affairs hospital in southern California, with the aim of contributing toward the understanding of Q fever in this region. Demographics, laboratory data, diagnostic imaging, risk factors, and treatment regimens were collected via a retrospective chart review of patients diagnosed with Q fever at our institution between 2000 and 2016. Cases were categorized as acute or chronic and confirmed or probable. The majority presented with an acute febrile illness (90%). There was a delay in ordering diagnostic serology from the time of symptom onset (acute cases, average 31.9 days; chronic cases, average 63 days), and 15% progressed from acute to chronic infection. Of the chronic cases, 22.2% had endocarditis, 22.2% had endovascular infection, and 11.1% had both endocarditis and endovascular infection. The geographic distribution revealed that 20% resided in rural areas. Of the cases of Q fever that died, death attributed to Q fever was associated with an average diagnostic delay of 65.5 days. Acute Q fever is underreported in this region largely because of its often nonspecific clinical presentation.

## INTRODUCTION

*Coxiella burnetii* is the obligate intracellular Gram-negative bacteria responsible for query fever (Q fever), which is known to cause acute and chronic infection in humans.^1^ The clinical manifestations of acute and chronic infection are often nonspecific and can be widely variable, which makes establishing the diagnosis challenging.^1,2^ Acute infection, although often nonspecific or even asymptomatic, can present with fever, myalgia, headache, or gastrointestinal symptoms, and chronic infection can range from granulomatous hepatitis, osteomyelitis, and pneumonia to endocarditis.^1^ Transmission is predominantly from direct contact with infected animals or their birth products, although multiple modes of transmission have been described in humans, including inhalation of contaminated wind-borne material from infected livestock and ingestion of unpasteurized dairy products.^3–8^ There have even been reports of sexual transmission of Q fever.^9,10^ The reservoirs for Q fever are thought to include both wild and domestic animals spanning many different species and may also include arthropods.^8,11,12^ However, most patients diagnosed with Q fever do not report any exposure to the known risk factors.^13^

Query fever was first described in 1935 in Queensland, Australia, and has since been reported in almost every country.^14^ Twelve years later, Frank Young described the first reported case of Q fever in Los Angeles County, where the dairy industry was flourishing and geographically concentrated.^15^ Subsequent studies conducted in southern California in the 1940s and 1950s, to better characterize the disease and its endemicity, associated Q fever with exposure to domestic livestock, mostly sheep, goats, and cattle.^16,17^ These studies reported that the largest number of serologically positive animals were from southern California, as compared with northern California, and suggested sheep and goats were primarily responsible for harboring the disease in the north, whereas cattle were predominantly responsible in the south.^18,19^ Not many studies on Q fever in southern California have been conducted recently, and to our knowledge, the most recent study was published in 2006 by Cone et al.,^20^ who described six cases of Q fever which presented over 32 years in the southern California desert.

We describe the clinical presentation, geographic distribution, and risk factors of 20 patients diagnosed with Q fever at the Veterans Affairs (VA) Loma Linda Healthcare System from 2000 to 2016.

## MATERIALS AND METHODS

### Study population and inclusion/exclusion criteria.

Cases of Q fever seen at our institution between 2000 and 2016 were identified by their International Classification of Diseases (ICD) 9 (083.0) and ICD 10 (A78) codes. Query fever cases originally diagnosed at a VA other than the Loma Linda VA were excluded. In addition, the microbiology laboratory queried their database for positive Q fever titers.

### Definition of acute and chronic disease.

Our case definition was adapted from the CDC National Notifiable Diseases Surveillance System, which categorizes cases of Q fever as acute or chronic and confirmed or probable based on the clinical criteria and supporting laboratory evidence.^21^ Table 1 defines our method of case classification.

**Table 1:** Case definitions of Q fever cases (adapted from CDC’s National Notifiable Diseases Surveillance System)^21^

	Acute Q fever	Chronic Q fever
Clinical criteria of infection	Fever and one or more of the following: rigors, severe retrobulbar headache, acute hepatitis, pneumonia, or elevated liver enzymes	Newly recognized culture-negative endocarditis (particularly in a patient with previous valvulopathy or compromised immune system); suspected infection of a vascular aneurysm or vascular prosthesis; or chronic hepatitis, osteomyelitis, osteoarthritis, or pneumonitis in the absence of other known etiology
Laboratory criteria	Laboratory confirmed	Laboratory confirmed
	Fourfold change in the IgG antibody titer to *Coxiella burnetii* phase II antigen by IFA between paired sera	IgG titer ≥ 1:800 to *C. burnetii* phase I antigen by IFA
	Laboratory supportive	Laboratory supportive
	Single IgG titer ≥ 1:128 to *C. burnetii* phase II antigen by IFA (phase I titers may be elevated as well) or	IFA IgG titer ≥ 1:128 and < 1:800 to *C. burnetii* phase I antigen
	elevated phase II IgG or IgM antibody reactive with *C. burnetii* antigen by ELISA, dot-ELISA, or latex agglutination	
Case classification	Confirmed acute Q fever	Confirmed chronic Q fever
	Laboratory-confirming serology with clinical evidence of infection	Clinical evidence of infection with laboratory confirmation
	Probable acute Q fever	Probable chronic Q fever
	Laboratory-supportive serology with clinical evidence of infection	Clinical evidence of infection with laboratory supportive serology
	Progression from acute Q fever to chronic Q fever	
	A case initially classified as confirmed or probable acute Q fever with subsequent development of laboratory-confirming evidence consistent with chronic Q fever and an identifiable focus of infection	

Q fever = query fever.

Acute Q fever typically presents itself as transient flu-like symptoms including fever, myalgias, severe headache, gastrointestinal symptoms, cough, and chest pain; however, presentation may also be asymptomatic.^21–23^ The clinical criteria of acute illness included acute fever and at least one of the following: rigors, severe retrobulbar headache, acute hepatitis, pneumonia, or elevated liver enzymes.^21^ Laboratory confirmation of acute Q fever cases included a 4-fold increase in sequential phase II IgG serologic titers.^21^ Laboratory-supportive evidence for acute Q fever included a single serologic phase II IgG titer of ≥ 1:128.^21^ Cases of probable acute Q fever were defined as a clinically compatible presentation associated with supportive laboratory evidence, and cases of confirmed acute Q fever included those that met the clinical criteria and were associated with laboratory-confirming serology.^21^

Chronic Q fever can be very specific and typically presents as culture-negative endocarditis or vascular aneurysms; however, early chronic Q fever can present as asymptomatic infection or with nonspecific symptoms.^1,2,24,25^ Clinical evidence supportive of chronic Q fever was defined as an identifiable focus of persistent infection. Laboratory confirmation of chronic Q fever cases was a phase I IgG titer ≥ 1:800.^21^ Laboratory-supportive evidence for chronic Q fever cases was defined as a single phase I IgG titer ≥ 1:128 but < 1:800.^21^

We defined progression of acute to chronic disease as a case initially classified as confirmed or probable acute Q fever with subsequent development of laboratory-confirming evidence consistent with chronic Q fever and an identifiable focus of infection.

Demographic information, laboratory data, diagnostic imaging, risk factors, and treatment regimens were obtained from a retrospective chart review.

In our study, we defined rural as per the U. S. Census Bureau, which states that areas designated as rural have population densities less than 1,000 people per square mile.^26^

## RESULTS

Of the initial 27 cases, two cases were excluded because the diagnosis of Q fever was made before January 1, 2000, and five cases were excluded because the diagnosis was made at an outside institution. This left 20 cases of Q fever diagnosed between 2000 and 2016 at the VA Loma Linda, CA.

Demographics, baseline characteristics, and initial laboratory values of Q fever cases are summarized in Table 2. Of the 20 patients who were diagnosed with Q fever at our institution, all patients were male with a mean age of 53.7 years (range 38–71 years) and predominantly white (65%). A wide range of medical comorbidities was noted, most commonly hypertension (45%), followed by substance abuse (40%) and diabetes (20%) (Table 2). Four patients (20%) had a history of vascular disease, which included three aortic aneurysms and one aberrant subclavian artery with descending aortic dissection. Three of these were repaired with vascular grafts. The majority of patients (14 cases, 70%) reported exposure to animals (Table 2), but only 35% reported contact with livestock (cattle, sheep, and goats). Eighteen of 20 (90%) patients presented with an acute febrile illness commonly together with other symptoms including headache, cough, hepatomegaly, and arthralgias or myalgias (Table 2).

**Table 2:** Characteristics of query fever cases

Characteristic	Total (*N* = 20)
Patient age, mean years (range)	53.7 (38–71)
Gender (%, male)	20 (100)
Race/ethnicity (%)	
White	13 (65)
Black	4 (20)
Asian/Pacific islander	2 (10)
Hispanic/Latino	1 (5)
Comorbidities (%)	
Hypertension	9 (45)
Alcohol or nicotine dependence	8 (40)
Valvular heart disease	6 (30)
Diabetes mellitus	4 (20)
Coronary artery disease	4 (20)
Aortic vascular disease	4 (20)
Chronic kidney disease	2 (10)
HIV/AIDS	2 (10)
Malignancy	1 (5)
Clinical characteristics	
Fever	18 (90)
Hepatomegaly or splenomegaly	11 (55)
Dyspnea or cough	11 (55)
Headache	9 (45)
Arthralgia or myalgia	8 (40)
Abdominal pain, nausea, or vomiting	5 (25)
Rash	3 (15)
Altered mental status	2 (10)
Chest pain	2 (10)
Ocular pain	1 (5)
Animal exposure	
Rodents	8 (40)
Dogs	6 (30)
Cats	5 (25)
Horses	5 (25)
Poultry	4 (20)
Other birds*	3 (15)
Goats	3 (15)
Swine	3 (15)
Sheep	2 (10)
Cattle	2 (10)
No exposure reported	6 (30)
Case classification	
Acute infection	14 (70)
Confirmed	11 (78)
Probable	3 (21)
Chronic infection, confirmed	3 (15)
Progression from acute to chronic disease	3 (15)
Acute_c_ to chronic_c_	2 (67)
Acute_p_ to chronic_c_	1 (33)

Acute_c_ = acute infection, confirmed; Acute_p_ = acute infection, probable; Chronic_c_ = chronic infection, confirmed.

* Other bird exposures included peacocks, canaries, cockatiels, and parrots.

Laboratory testing revealed normal hematology for all but five cases that exhibited thrombocytopenia (Table 3). Most cases demonstrated a mild elevation of aspartate aminotransferase (AST) and alanine aminotransferase (ALT), although four cases had either an ALT or AST > 200 U/L (Table 3). Thirteen of 20 (65%) cases had an elevated erythrocyte sedimentation rate with a mean of 110.2 mm/hour (Table 3).

**Table 3:** Laboratory characteristics of query fever cases

Laboratory characteristics (reference range)	Result mean (range)
Hematology	*n* = 20
White blood cell (4.0–10 × 10^9^/L)	9.9 (3.8–18)
Hematocrit (40–53%)	38.1 (27–49.6)
Platelets (150–350 × 10^9^/L)	221.7 (42–554)
Miscellaneous hematology	*n* = 13
Erythrocyte sedimentation rate (0–15 mm/hour)	110.2 (6–115)
Chemistry	*n* = 20
Alanine aminotransferase (0–35 U/L)	101.9 (12–444)
Aspartate aminotransferase (0–35 U/L)	75.5 (12–229)

**Figure 1.**
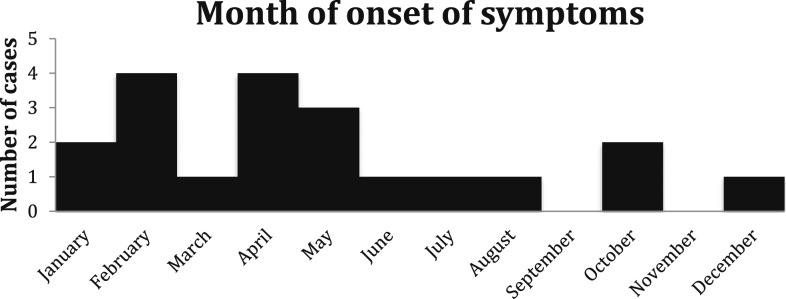
First month of symptom onset for query fever cases between 2000 and 2016.

Symptom onset was most frequent during the spring and winter seasons (75%) (Figure 1). The geographic distribution of cases favored urban areas, given only 20% of our cases resided in rural areas.

Seventeen of 20 (85%) cases were diagnosed as a result of infectious disease consultation and follow-up. Three cases did not involve infectious disease consultation. To our knowledge all of these were acute cases that resolved with treatment (there were no follow-up titers for these cases). The delay in ordering diagnostic serology from the time of symptom onset ranged from 4 days to 168 days (Table 4). Cases of acute Q fever that did not progress to chronic disease had an average delay of 31.9 days from the symptom onset to the date that diagnostic titers were ordered, whereas the average for those that progressed to chronic Q fever was 72 days (Table 4). The cases with noncardiac endovascular infection had the longest delay in ordering diagnostic titers, on average, 109.5 days (Table 4). Cases that involved infectious disease consultation had an average time from consultation to ordering diagnostic titers of 4.5 days (range 0–41 days) (Table 4).

**Table 4:** Time from symptom onset to diagnostic Q fever titers

	Mean time (days) from the symptom onset to diagnostic titers (range)
Acute Q fever	31.9 (7–168)
Progression from acute to chronic infection	72 (14–122)
Chronic Q fever	
Endocarditis	65.5 (51–80)
Noncardiac endovascular infection	109.5 (97–122)
Endocarditis and noncardiac endovascular infection	14 (14)
Infectious disease consultation	4.5 (0–41)
Death (all cases)	55.9 (14–168)
Death attributed to Q fever	65.5 (51–80)

Q fever = query fever.

The treatment course of each case is detailed in Table 5. Nineteen of 20 (95%) patients were treated with a minimum of 10 days of doxycycline. One case elected for hospice care for medical issues unrelated to the diagnosis of Q fever and did not receive treatment (Table 5). Four (20%) patients are presently on long-term treatment ranging from 11 months to 2 years. Three of four (75%) patients on chronic therapy have already completed 24 months of therapy (Table 5). Four of five (80%) cases of endocarditis and noncardiac endovascular infection were treated with combination therapy (doxycycline and either hydroxychloroquine or rifampin), whereas one case of endocarditis was initiated on doxycycline, but the patient expired before combination treatment could be initiated.

**Table 5:** Summary of demographic, clinical features, and outcomes of Q fever cases

Patient no.	Age (years)	Clinical presentation	Animal exposure	Date of symptom onset	Date of first titer	Case classification	Treatment (duration)	Outcome	Death (date)	Q fever contribution to death
1	50	Febrile illness	Yes	April 15, 2000	May 20, 2000	Acute_p_	DOX + RIF (8 weeks)	Resolved	–	–
2	49	Febrile illness	Yes	February 25, 2001	March 9, 2001	Acute_c_	DOX + RIF (3 weeks)	Resolved	–	–
3	38	Febrile illness	Yes	February 4, 2004	March 26, 2004	Chronic_c_	DOX + CIP + CLQ + AZI + RIF (2005–2011); HCQ (2004–2010)	Endocarditis	Yes (January 3, 2011)	Yes
4	44	Febrile illness	No	February 4, 2004	May 5,2004	Acute_p_	DOX + RIF (30 days)	Resolved	Yes (August 13, 2017)	No
5	69	Febrile illness with dyspnea	No	May 11, 2004	May 27, 2004	Acute_p_	DOX (2 weeks)	Resolved	Yes (January 30, 2012)	No
6	51	Febrile illness	Yes	May 23, 2005	June 7, 2005	Acute_c_	DOX (2 weeks)	Resolved	–	–
7	58	Febrile illness	Yes	July 15, 2005	August 1, 2005	Acute_c_	DOX (2 weeks)	Resolved	Yes (November 17, 2016)	No
8	42	Febrile illness, headache, and low back pain	Yes	January 9, 2006	January 23, 2006	Acute_c_	DOX (2 weeks)	Resolved	Yes (August 21, 2017)	Unknown
9	57	Febrile illness with lymphadenopathy	Yes	December 28, 2006	January 11, 2007	Acute_c_ to chronic_c_	DOX (2 weeks in 2007, and 2015–2016) and HCQ (2015–2016)	Endocarditis and endovascular infection (mycotic aortic pseudoaneurysm with rupture)	Yes (December 23, 2016)	Unknown
10	49	Febrile illness	No	February 8, 2007	February 15, 2007	Acute_c_	DOX (3 weeks)	Resolved	–	–
11	53	Febrile illness	Yes	April 9, 2010	April 20, 2010	Acute_c_	DOX (10 days)	Resolved	–	–
12	58	Febrile illness and retro-orbital eye pain	Yes	May 7, 2016	May 18, 2016	Acute_c_	DOX + HCQ (November 2, 2017–present, anticipated 18 months)	Persistently elevated titers	–	–
13	48	Chest pain, cough, and anorexia	Yes	April 1, 2016	July 7, 2016	Chronic_c_	DOX + HCQ (July 25, 2016–present, anticipated 2 years)	Endovascular infection (aberrant subclavian artery with carotid–subclavian bypass homograft)	–	–
14	64	Febrile illness with groin pain	No	April 7, 2014	August 7, 2014	Acute_c_ to chronic_c_	DOX + HCQ (2014–2018)	Endovascular infection (infrarenal abdominal aortic aneurysm with endovascular repair and common iliac stent)	Yes (July 28, 2018)	Unknown
15	58	Febrile illness with severe migrating necrotizing pneumonia and eosinophilia	Yes	October 24, 2011	October 28, 2011	Acute_c_	DOX (November 18, 2011–August 21, 2017)	Persistently elevated titers	Yes (August 21, 17)	No
16	68	Febrile illness	Yes	February 10, 2016	February 17, 2016	Acute_c_	DOX (February 17, 2016–present)	Persistently elevated titers	–	–
17	50	Febrile illness	No	June 2, 16	June 14, 2016	Chronic_c_	DOX, HCQ (June 22, 2016–present)	Granulomatous hepatitis	–	–
18	48	Febrile illness with abdominal pain	Yes	August 1, 2007	January 16, 2008	Acute_c_	No treatment	Hospice	Yes, on hospice (September 30, 2009)	No
19	48	Febrile illness, confusion, palpitations, and knee pain	Yes	March 10, 2008	April 17, 2008	Acute_c_	DOX and LEV (both stopped because of side effects after 11 months)	Persistently elevated titers	Yes (October 23, 2015)	Unknown
20	71	Dyspnea with altered mental status	No	October 1, 2010	December 20, 2010	Acute_p_ to chronic_c_	DOX + RIF (8 weeks)	Endocarditis and possible Q fever meningoencephalitis	Yes (February 21, 2011)	Yes

Acute_c_ = acute infection, confirmed; Acute_p_ = acute infection, probable; AZI = azithromycin; Chronic_c_ = chronic infection, confirmed; Chronic_p_ = chronic infection, probable; CIP = ciprofloxacin; CLQ = chloroquine; DOX = doxycycline; HCQ = hydroxychloroquine; LEV = levofloxacin; RIF = rifampin; Q fever = query fever.

Table 6 presents the clinical outcomes of the 20 patients diagnosed with Q fever. Of the 14 patients who presented with acute Q fever, five (35.7%) exhibited persistently elevated titers without evidence of chronic infection and three (21.4%) progressed to chronic infection. Two cases had definite endocarditis by Duke criteria (culture negative), two developed noncardiac endovascular infection from preexisting vascular conditions, and one case had both endocarditis (definite by Duke criteria and culture negative) and noncardiac endovascular infection. One case of chronic Q fever had biopsy-proven granulomatous hepatitis. Query fever was thought to be a contributing factor in two of the 11 deaths to date (Table 6). Two cases where death was thought related directly to Q fever had an average of 65.5 days delay in ordering diagnostic titers.

**Table 6:** Clinical outcomes of Q fever cases

Outcome	Number of cases (%)
	*N* = 20
Acute disease with resolution	9 (45)
Acute disease with persistently elevated titers	5 (25)
	*N* = 6
Chronic infection	
Endocarditis	2 (22.2)
Noncardiac endovascular infection	2 (22.2)
Endocarditis and noncardiac endovascular infection	1 (11.1)
Hepatic infection	1 (11.1)
	*N* = 11
Death	11 (55)
Q fever contribution	2 (10)
No clear contribution of Q fever	5 (45.5)
Unknown etiology	4 (36.4)

Q fever = query fever.

## DISCUSSION

Our study showed a higher proportion of chronic Q fever cases and a higher case fatality rate than the reported national average. A large study conducted in the United States using surveillance data collected for the CDC reported a case fatality rate of 2%.^13^ Our case fatality rate was 10% over the 17 years of this case series. The mortality of acute Q fever is very low; however, chronic Q fever is associated with a high mortality rate, especially if untreated.^2,27^ The delay in ordering diagnostic titers from the date of symptom onset may have contributed to the higher proportion of chronic Q fever cases and the higher case fatality rate. Efforts to decrease the delay in diagnosis are crucial because subsequent development of chronic Q fever is associated with a poor prognosis.^3^ A more timely diagnosis of Q fever with earlier initiation of treatment may result in fewer hospitalizations and fewer severe complications.^27^ Our data also suggest that early involvement of infectious disease consultation was associated with earlier diagnosis.

Acute Q fever is underdiagnosed and underreported in the United States.^11,22,28^ The literature reports a large percentage of acute Q fever cases as asymptomatic infection; however, our study did not capture this population as all our cases were symptomatic.^1,2,29^ The national seroprevalence of Q fever in the United States was reported to be 3.1%, between 2003 and 2004, which is higher than expected based on the number of cases reported to the CDC.^28^ A large study conducted by Dahlgren et al.,^22^ which is the most comprehensive summary of Q fever trends in the United States to date, estimated that for every reported case of Q fever, 13 cases go unreported. Acute Q fever represented 70% of our cases, compared with the national data from the CDC which reports that acute Q fever cases comprise 75–90% of reported cases annually.^30^ We believe that acute Q fever is an under-ascertained infection because individuals who lack symptoms or have mild or self-limiting symptoms do not present for health-care evaluation.

Chronic Q fever is mostly seen in male patients with valvulopathy, but remains a rare disease.^30^ Reported cases of acute Q fever rarely progress to chronic infection (< 5%);^1^ however, 21.4% of our acute cases demonstrated progression to chronic infection. The diagnosis and management of chronic Q fever remain challenging. There is a lack of international consensus regarding the distinction between acute and chronic Q fever, and there is no single management strategy to date.^2,31^ Prior studies conducted in France found the most common manifestation of chronic Q fever was endocarditis.^32^ By contrast, data from a recent large outbreak in the Netherlands found that the predominant manifestations of chronic Q fever are infected aneurysms and vascular prostheses.^25^ It remains unclear which is the more common manifestation in the United States; however, it appears to differ geographically worldwide.^31^ Query fever endocarditis is a uniformly fatal condition if untreated and is associated with a 10-year mortality rate of 19% even in patients who receive treatment.^1,33^ All of our endocarditis cases expired within 10 years of diagnosis regardless of treatment, and both cases where Q fever was thought to be a contributor toward death were endocarditis cases. According to the literature, most vascular infections involving *C. burnetii* involve preexisting lesions of the aorta.^1^ Four of our cases carried a previous diagnosis of aortic vascular disease, and of these, three developed noncardiac endovascular infections. No microorganism was isolated from these cases, which is consistent with the findings of Fournier et al.,^32^ who only demonstrated isolation of a microorganism in 25 of 163 patients. This group also had the longest delay from symptom onset to ordering diagnostic titers (an average of 109.5 days).

Although serology is the first-line diagnostic approach, several methods have been shown to assist with the diagnosis of *C. burnetii* infection.^31^ Detection of *C. burnetii* DNA by polymerase chain reaction (PCR) in clinical samples has the advantage of being able to detect the organism before seroconversion and has been shown to be a strong indicator of persistent infection.^2,31^ Studies by Fenollar et al.^34^ have also proposed a rapid nested-PCR to assist in establishing an early diagnosis of chronic Q fever. The quantification of IgG anticardiolipin (aCL) antibody levels has been suggested to directly correlate with a high positive predictive value for disease progression from acute Q fever to endocarditis.^35,36^ Only one of our cases had quantification of IgG aCL antibody levels, which were low positive, and went on to develop persistently elevated titers without evidence of endocarditis to this date. It has also been suggested that IgG aCL antibody levels were an earlier and more predictive determinant of progression to endocarditis than Q fever serology.^36^ Thus, obtaining aCL antibody levels at the diagnosis of acute Q fever should be given consideration and increased provider education remains important. Finally, the 18 F-FDG PET/CT-scan has demonstrated the ability to localize persistent foci of infection in chronic Q fever and increase the detection of Q fever endocarditis in patients without valvular lesions on echocardiography.^31,37^

Our cases resided in Riverside and San Bernardino Counties of the southern California desert, where Q fever is endemic and enzoonotic. The region is characterized by mild winters, very warm spring and summer seasons, and gusty seasonal winds, the “Santa Ana winds,” occurring typically between October and March. The ability of Q fever to be carried long distances via dust and wind has been characterized,^6,7^ and thus, southern California provides an ideal climate for the propagation and spread of the disease. It has been recently reported that across the United States, the most common occupations among cases of Q fever are ranchers and military personnel, most of whom reported travel to the Middle East.^13^ However, only one of our cases (number 13) reported travel to Afghanistan 4 years before diagnosis with Q fever. The seasonality of Q fever symptom onset has been reported mostly during the spring and early summer months, with a peak during the months of April and May.^30^ This study also saw a peak in April and May, and a winter peak in February. The etiology of the February peak is unclear; however, the April and May peaks likely coincide with the birthing season for many domesticated animals.

From the epidemiologic studies of California in 1948–1949, Riverside and Orange counties exhibited the highest percentage of positive titers in cattle when compared with the rest of the state.^18^ Other serologic surveys conducted in neighboring counties found that the seroprevalance of Q fever among dairy herds remained high 20 years later.^19^ Although 30% of cases did not report any animal contact, it has been demonstrated that living around areas where animals are kept is a risk factor for the disease.^20^ Although the continued presence of the dairy industry in these counties is interesting to note, it is difficult to use this information to draw definitive associations without serology from the animals. The Netherlands recently experienced the largest outbreak of Q fever to date, about 4,000 cases from 2007 to 2010, in which dairy goats were found to be the main source of disease.^25^ In addition, it has been demonstrated that preventive veterinary measures, such as routine vaccination to build herd immunity, can reduce the environmental spread of *C. burnetii* and, subsequently, moderate the transmission to humans.^25^

Our study had several limitations, the first being the retrospective, single-institution model of this observational case series. The application of our data may be limited, given all data were collected from a single institution that serves military veterans who are predominantly male. However, some may feel that this is a strength, given the similar testing methodologies and consistent catchment of patients. Four patients expired at non-VA hospitals, and medical records surrounding these circumstances were not available for review at the time of this study. The serologic method used to determine the titers was predominantly immunofluorescence assay (IFA); however, testing was performed by several different reference laboratories across the United States. DNA detection confirmation testing was not performed on any of our cases, and none of our cases demonstrated isolation of the organism by culture or immunohistochemical methods. Low-level Q fever titers may not have been detected by the microbiology laboratory query, and cases may not have been identified if providers did not link the diagnosis to ICD 9 or ICD 10 codes.

In conclusion, we recommend obtaining Q fever serology in all patients residing in endemic areas who present with a febrile illness and negative blood cultures.^38^ In our experience, involving infectious disease consultation early in the clinical course appears to shorten the time from symptom onset to diagnosis. Given the severity of Q fever endocarditis and noncardiac endovascular infections, systematic detection of *C. burnetii* with PCR and screening for valvular and vascular risk factors has been recommended.^23,24^ Reporting Q fever is reliant on the awareness of the disease and its endemic nature and a high threshold of suspicion by clinicians.^11^ Increasing physician awareness and, therefore, reporting of the disease, mandatory reporting of animal infection, and systematic seroprevalence studies in humans and animals would provide important information for the prevention of disease.^11^ Further epidemiologic studies and increased surveillance may also clarify whether livestock from existing dairy farms remain a potential reservoir for the disease in our geographic area, and provide important information regarding Q fever trends and disease outbreaks in our region.
